# Seasonality and outbreak of a predominant *Streptococcus pneumoniae *serotype 1 clone from The Gambia: Expansion of ST217 hypervirulent clonal complex in West Africa

**DOI:** 10.1186/1471-2180-8-198

**Published:** 2008-11-17

**Authors:** Martin Antonio, Ishrat Hakeem, Timothy Awine, Ousman Secka, Kawsu Sankareh, David Nsekpong, George Lahai, Abiodun Akisanya, Uzochukwu Egere, Godwin Enwere, Syed MA Zaman, Philip C Hill, Tumani Corrah, Felicity Cutts, Brian M Greenwood, Richard A Adegbola

**Affiliations:** 1 Bacterial Diseases Programme, Medical Research Council Laboratories, Banjul, The Gambia; 2 London School of Hygiene and Tropical Medicine, London, UK

## Abstract

**Background:**

*Streptococcus pneumoniae *serotype 1 causes > 20% of invasive disease, among all age groups combined, in The Gambia. In contrast, it is rarely detected in carriage studies. This study compares the molecular epidemiology of *S. pneumoniae *serotype 1 causing invasive disease in The Gambia between 1996 and 2005 to those carried in the nasopharynx between 2004 and 2006.

**Results:**

A total of 127 invasive and 36 nasopharyngeal carriage serotype 1 isolates were recovered from individuals of all age groups and were analyzed by serotyping, antibiotic susceptibility testing and MLST. MLST analysis revealed 23 different sequence types (STs), 18 of which were novel. The most prevalent clone among the 163 isolates was ST618 (70.5%), followed by ST3575 (7.4%), ST2084 (2.5%) and ST612 (2.5%). A single ST (ST618), previously shown to belong to the ST217 hypervirulent clonal complex, was frequent among carriage (61.1%) and invasive (72.7%) serotype 1 isolates. ST618 causing both paediatric and adult disease peaked annually in the hot dry season and caused outbreak in 1997 and 2002.

**Conclusion:**

For over a decade, isolates of ST618 have been the dominant lineage among serotype 1 carriage and disease isolates circulating in the Gambia. This lineage shows similar epidemiological features to those of the meningococcus in the African meningitis belt being able to cause outbreaks of disease

## Background

*Streptococcus pneumoniae *consists of at least 91 different serotypes, the majority of which rarely cause disease; two serotypes (1 and 5) cause > 30% of invasive disease, among all age groups combined, in The Gambia [1]. Serotype 1 has the potential to cause epidemics – it was recently a cause of outbreaks of meningitis in Burkina Faso and Northern Ghana [2,3]. It has also been associated with outbreaks in crowded or closed communities [4-6]. In contrast, it is only rarely detected in carriage studies [7]. The available, licensed pneumococcal conjugate vaccine (Prevenar^®^) contains seven of the 91 pneumococcal serotypes and does not contain conjugates of serotypes 1 and 5 [8]. A trial of a 9-valent pneumococcal conjugate vaccine in a rural setting in The Gambia showed protective efficacy against radiological pneumonia (37%) and all invasive pneumococcal disease (50%) and reduced all cause hospitalisations and mortality by 15% and 16% respectively [9]. Although the 9-valent vaccine contained serotype 1 glycoprotein, pneumococci of this serotype caused a small number of cases of invasive disease, with no reduction in vaccinated children [9], A combined analysis of the efficacy of serotype 1 vaccines against invasive pneumococcal disease in The Gambia and South Africa showed no evidence of protection against this serotype (27%, 95% CI-120, 76), although these were the only two trials that have assessed this and the total numbers of cases were small [10] Genotyping analysis showed that all but one (ST3336) of the eight *S*. *pneumoniae *serotype 1 isolates responsible for cases of invasive disease in The Gambian conjugate vaccine trial exhibited ST618 [11]. To understand the invasive potential of *S*. *pneumoniae *serotype 1 ST618 lineage, we investigated the molecular epidemiology and invasive capacity of *S*. *pneumoniae *serotype 1 isolates from rural and urban Gambia obtained during the period 1996–2006, comparing isolates obtained from asymptomatic nasopharyngeal carriers or cases of invasive pneumococcal disease in all age groups.

## Methods

### Invasive and carriage isolates

The *S. pneumoniae *serotype 1 isolates used in this study were collected from four of the five geographically diverse regions including the Kombos (urban, rural, and peri-urban), Western (urban), Central (rural) and the Upper River (rural) Regions of The Gambia between 1996 and 2006 (Table 1). *S*. *pneumoniae *serotype 1 carriage isolates were recovered from nasopharyngeal swab specimens of healthy Gambians as previously described [7,12]. A diagnosis of invasive disease was based on isolation of the pneumococcus from blood, cerebro-spinal fluid (CSF), lung aspirate, or a combination of sites as previously described [1,9]. Isolates were stored at -70°C in 15% glycerol broth before further testing. When more than one isolate was obtained during a single episode of illness, for example from blood and CSF, multiple isolates have been included in the analysis only if they differed by MLST and/or antibiotic susceptibility pattern. Only one child had two episodes of illness. Details of how children were enrolled, followed up and investigated have been described previously [1,7,9,12,13]. Written consent was obtained from the parents and the trial was approved by the Joint Gambia Government and MRC Ethics Committee and Ethics Committees at the London School of Hygiene & Tropical Medicine and the World Health Organization.

**Table 1:** Provenance of *S. pneumoniae *isolates used in this study

**Surveillance Method^‡‡^**	**Geographic Area^‡‡^**	**Year of study**	**Age range studied**	**Invasive, n (%)**	**Carriage, n (%)**	**Reference**
Routine hospital surveillance^†^	Kombos, WR	1996–2003	1 day-78 years	93(73.2)	-	[1], this study
Treatment trial*	URR & CRR	2004–2005	4–46 months	-	7(19.4)	This study
Hib vaccine trial**	WR	1997–2002	< 6 years	26(20.5)	-	[9]
Pneumococcal vaccine trial*	URR & CRR	2000–2004	2–29 months	8(6.3)	-	[13]
Pneumococcal vaccine trial*	URR & CRR	2003–2005	12–23 months	-	14(38.9)	This study
Carriage study	WR	2004–2006	0.5–70 years	-	15(41.7)	[7,12]
						
**Total**	-	-	-	127 (100)	36 (100)	

^†^Isolates were collected from patients who presented routinely to the MRC outpatient clinic in Fajara, The Kombos [1].

*Isolates were collected during prospective studies or trials of vaccines or pneumonia case management regimens with a standardized surveillance system for invasive pneumococcal disease. Subjects may have been randomised to receive the nine-valent pneumococcal vaccine (5 isolates were from 4 PCV-vaccinated children and 3 isolates were from 2 non-PCV-vaccinated children) [9].or high dose amoxicillin [20], unpublished].

**Subjects were investigated for invasive bacterial disease during the course of an Hib vaccine effectiveness study [13].

n, refers to the number of serotype 1 isolates

^‡‡^Kombos (urban, rural, and peri-urban), WR; Western Region (urban), CRR; Central River Region (rural) and URR; Upper River Region (rural).

Notes: There is one year overlap between invasive and carriage isolates

### *S. pneumoniae *isolation, serotyping and antimicrobial susceptibility

*S. pneumoniae *isolation, serotyping and antimicrobial susceptibility testing were performed as described previously [1,7,9]. When more than one isolate was obtained during a single episode of illness, for example from blood and CSF, multiple isolates were included in the analysis only if they differed by MLST and/or antibiotic susceptibility pattern. The MRC microbiology laboratory submits to the external quality assurance programme of the United Kingdom National External Quality Assessment Service [14].

### Multi locus sequencing typing (MLST)

*S. pneumoniae *isolates were streaked on blood agar and incubated at 37°C for 18 hours. A single colony from each isolate was picked, restreaked and incubated at 37°C for 18 hours. Genomic DNA templates were prepared from a loopful of bacteria as described in the manufacturer's instructions (Qiagen Genomic DNA Kit, UK). MLST was performed as described [15]. The seven genes targeted are *aroE, gdh, gki, recP, spi, xpt *and *ddl*. Amplifications for all genes were carried out with approximately 0.2 μg DNA template, 250 μM (each) deoxynucleoside triphosphates, 2.5 mM MgCl_2_, 25 pmol of primers, and 1 U of Taq polymerase (Qiagen) in a 25 μl reaction mixture. PCR cycling conditions were a 10 min hold at 94°C, followed by 34 cycles of 94°C for 1 min, 55°C for 1 min, and 72°C for 1 min, and a final extension at 72°C for 5 min. 2 μl of reaction mixtures were separated by 1.0% agarose gel electrophoresis and visualized with ethidium bromide staining and UV illumination with a gel documentation system (Gel Doc 2000; Bio-Rad, UK).

PCR products were purified and DNA sequenced on both strands in-house in our Core Sequencing Facility in Fajara, The Gambia. Sequences were edited and complementary sense and antisense fragments were aligned using the Laser Gene DNA star 7.1 software. The sequences were submitted to the MLST database website [16] and assigned existing or novel allele type numbers and sequence type numbers defined by the database. This multi microorganism database defines a novel allele type if it contains one or more nucleotide changes from existing allele sequences. Composite sequence types (STs) are assigned based on the set of allele types derived from each of the seven loci. STs were analyzed for relatedness using the eBURST v3 program [17]. Cluster analysis of allelic profiles was performed using a categorical coefficient and a graphic method called a minimum spanning tree with Bionumerics software (version 4.0; Applied Maths, Sint-Martens-Latem, Belgium).

### Estimation of the invasiveness of clones

An empirical odds ratio (OR) was calculated with 95% CI using stata to compare the probability of invasive disease following colonisation with varying clones. An OR > 1 indicates an increased probability to cause invasive disease.

## Results

### Clinical epidemiology

**Figure 1:**
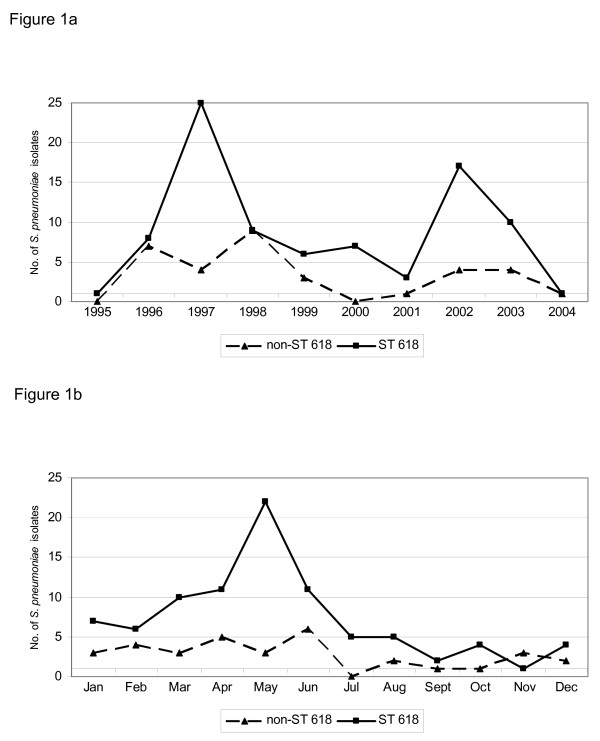
**Numbers of laboratory-confirmed serotype 1 pneumococcal isolates obtained from patients with invasive disease by (a) year and (b) month in the study areas of The Gambia from 1996–2006.** Bars indicate the total number of serotype 1 pneumococcal isolates; squares those belonging to ST 618 and diamonds those belonging to non-ST618 clones.

Invasive *S. pneumoniae *isolates were recovered from specimens of blood (75%), lung aspirate (14%), CSF (7%), knee aspirate (3%) and abdominal aspirate (1%). The median age of the patients with invasive disease was 6.0 years (IQR 2.3–30 years). The number of pneumococcal isolates obtained from patients with invasive disease varied from year to year (fig 1a) with peaks in 1997 and 2002. Yearly fluctuations were due largely to changes in the number of pneumococci of ST618 obtained. Pneumococcal isolates were obtained most frequently at the end of the hot dry season (May), this seasonal peak being accounted for largely by an increase in isolation of pneumococci of ST618 (fig 1b).

The median age of the individuals from whom the 36 carried isolates were obtained was 7.85 years (IQR 0.5–71 years).

### Antibiotic susceptibility among carried and invasive isolates

All invasive and carriage isolates were susceptible to chloramphenicol, penicillin and cefotaxime, whilst susceptibility to cotrimoxazole was 57% and 66% for carriage and invasive isolates respectively (p = 0.028). Susceptibility to tetracycline was 50% and 79% for carriage and invasive isolates respectively (p = 0.0003).

### Clonal relationship among invasive and carriage isolates and OR for invasiveness

The genetic cluster of the study isolates by BOX-PCR showed many instances in which the same ST produced different BOX-PCR patterns indicating heterogeneity within ST (data not shown).

MLST showed that there were 23 unique sequence types (ST) in the collection (8 from carriage isolates only, 10 from invasive isolates only and 5 from both). Eighteen of these STs (78.2%) were novel, i.e. not found in the *S. pneumoniae *MLST database [16]. The most prevalent clone among the 163 isolates was ST618 (70.5%), followed by ST3575 (7.4%), ST2084 (2.5%) and ST612 (2.5%). ST 618 represented 22 of 36 (61.1%) carried isolates and 93 of 128 (72.7%) invasive isolates (Table 2).

**Table 2:** Sequence type distribution (ST) of *S. pneumoniae *isolates used in this study

				**Allele profiles**				**No of isolates**		**Total no. of Isolates**
	
**ST**	**aroE**	**gdh**	**gki**	**recP**	**spi**	**xpt**	**ddl**	**Carriage**	**Invasive**	
ST217	10	18	4	1	7	19	9	1	5	6
ST612	10	18	4	1	7	19	31	1	3	4
ST 618	13	8	4	1	7	19	14	22	93	115
ST1331	13	8	4	1	7	19	9	1	1	2
ST2084	13	8	4	2	7	19	14	2	2	4
ST3570	7	18	4	1	7	19	31	1	0	1
ST3571	7	13	101	4	36	1	74	1	0	1
ST3574	13	8	4	1	7	19	19	2	0	2
ST3572	7	2	4	4	7	17	19	1	0	1
ST3573	13	8	4	15	7	19	14	0	1	1
ST3574	13	8	4	1	7	19	19	0	1	1
ST3575	10	191	4	1	7	19	9	0	12	12
ST1336	13	8	4	1	7	19	161	0	1	1
ST3576	5	17	4	4	6	1	17	0	1	1
ST3577	10	8	4	1	7	19	14	0	1	1
ST3578	1	8	193	5	6	58	8	0	1	1
ST3579	13	8	4	5	7	250	14	0	2	2
ST3580	10	13	53	1	72	19	31	0	1	1
ST3581	13	8	4	115	7	19	14	0	2	2
ST3582	12	43	194	1	181	49	31	1	0	1
ST3407	23	189	4	12	43	4	74	1	0	1
ST3583	2	20	4	38	27	88	6	1	0	1
ST3329	7	13	4	8	6	20	18	1	0	1

Total								36 (100)	127 (100)	163

Novel alleles are underlined.

A high prevalence of ST 618 was linked to the dramatic increase in the isolation rate for serotype 1 pneumococci in The Gambia in 1997 (figure 1). The odds ratio of ST 618 causing invasive disease relative to non-ST 618 clones was 1.74 (95%CI, 0.80–3.78; *P *= 0.162. In addition, there was no significant association between ST 618 and disease outcome (death or discharge from hospital). The odds ratio of ST 618 causing death relative to non-ST 618 was 0.67 (95% CI, 0.16–3.31; *P *= 0.504) (data not shown).

**Figure 2:**
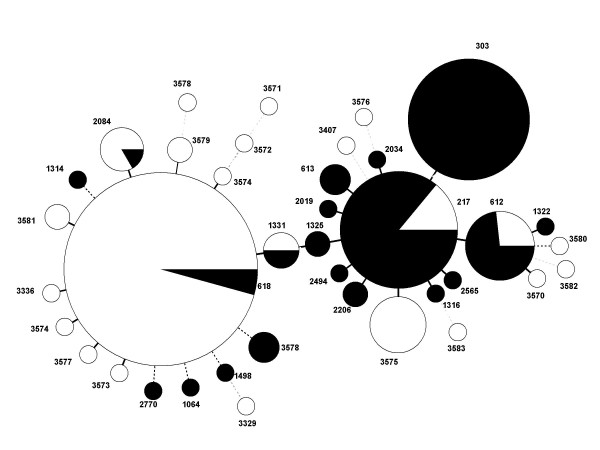
**Clustering of STs by use of the minimum spanning tree.** Each circle represents an ST. The area of each circle corresponds to the number of isolates. Thick, short, solid lines connect single-locus variants and thin, longer, solid lines connect double-locus variants. Unshaded (white) portions represent Gambian STs and shaded (black) portion represents ST found in the rest of the world.

An eBURST analysis that included the STs from this study and all serotype 1 isolates for which STs was available in the MLST database was performed using the stringent 6/7 identical loci definition. eBURST groups these ST into one major (ST217) and two minor (ST618 and 303) clonal complexes (data not shown). The results of cluster analysis of all the serotype 1 STs using the minimum spanning tree is shown in figure 2. Five of the 23 STs found in The Gambian isolates (ST217, ST612, ST618, ST1331 and ST2084) have been previously described [16] and are indicative of lineage B isolates found predominantly in Africa and Israel [18].

### Discovery of new MLST alleles

We discovered six new alleles including one *gdh *(191), two *gki *(193,194), one *recP *(115) one, *spi *(181), and one *xpt *(250).

## Discussion

The high prevalence of ST618 among serotype 1 isolates from the Gambia in both invasive and carriage isolates suggest that this clone has spread widely in the country. The increase in serotype 1 disease seen in The Gambia in 1997 and to a lesser extent in 2002 was due primarily to cases caused by pneumococci of ST618. Because surveillance procedure varied during the course of the study, apparent variations in incidence from year to year must be treated with caution but the data presented in figure 1a suggests that there was significant outbreak of ST618 invasive pneumococcal disease in The Gambia in 1997. The number of isolates varied substantially by month of year and this seasonal pattern is unlikely to have been confounded by differences in sampling techniques from year to year. The increase in isolates observed at the end of the dry season was due largely to ST618; isolates of other non-ST618 and non-serotype 1 pneumococci (data not shown) showed little variation from month to month. The end of the dry season is the time of the year when meningococcal epidemics reach their peak in the African meningitis belt [19]. This suggests that serotype 1 pneumococci of ST618 share some characteristics with meningococci that make them more adapted for spreading in the face of environmental extremes. This finding is in agreement with the results of other studies in Ghana and Burkina Faso [2,3]. Recent outbreaks of meningitis in Ghana, Burkina Faso and Niger were associated with serotypes 1 ST618 and its clonal complex ST217 [2,3]. However, in The Gambia, ST618 was associated more with bacteriemia and pneumonia than with meningitis. In our study invasive serotype 1 isolates were obtained using passive (health-facility-based) surveillance methods and some invasive serotype 1 isolates could have been missed. We have recently started a large surveillance study of invasive pneumococcal disease in The Gambia using standardized techniques which may provide more information on the relative virulence of different genotypes.

A possible limitation of our study is that invasive and carriage isolates were collected during different but overlapping time periods (invasive isolates 1996–2004; carriage isolates 2003–2006) although both sources of isolates encompassed persons living in a wide geographical area, in different regions of The Gambia. Invasive isolates were collected during trials of vaccines or pneumonia case management regimens that used standardized surveillance for invasive pneumococcal disease among children enrolled in the trials, and from patients of all ages who presented routinely to the MRC hospital in Fajara. In addition, the laboratory methods employed for the isolation of pneumococcal varied over the years. Nonetheless, any potential effect of these differences in surveillance methods should have affected all STs and not just ST 618.

The number of serotype 1 carriage isolates studied was small because this serotype is only rarely recovered during carriage studies [7]. Despite this we have been able to show convincingly that serotype 1 pneumococci of ST618 are more common in both carriage and invasive disease than serotype 1 pneumococci of other STs. The factors that determine the transition from carriage to invasive pneumococcal disease are not well understood and it is not known why pneumococci of serotype 1 show a predilection for causing invasive disease. A recently published study based on a world-wide, diverse collection of 166 serotype 1 isolates was restricted to invasive isolates [18]; the authors could not therefore calculate the invasive potential of these genotypes. In our study, the OR of ST618 for causing invasive disease relative to non-ST618 serotype 1 isolates was not significant 1.74 (95%CI, 0.80–3.78; P = 0.162) and there was no association between genotype (ST) and disease outcome. Interestingly, an eBURST analysis has previously shown that ST618 belongs to the ST217 clonal complex that caused an outbreak of meningitis in Ghana. [2]. In addition to ST618, the other STs found in this study included ST 3575, ST217 ST612, ST1331 and ST2084 (Table 2). ST 3575, a novel clone was only isolated from sporadic invasive sources in the Kombos region in The Gambia between 1996–1999 with 20% case fatality (data not shown). All these STs belong to the lineage B ST217 clonal complex [18] but they were not significantly associated with elevated odds of invasive disease in this study. Lineage B is common in Africa and Israel, and distinct from lineage A and C. Lineage A isolates and ST have been found exclusively in Europe and North America and lineage C isolates have come mainly from Chile [18].

We found greater susceptibility to cotrimoxazole and tetracycline antibiotics among invasive isolates than among carriage serotype 1 isolates. It is encouraging that in The Gambia, pneumococcal serotype 1 isolates are still sensitive to chloramphenicol, which remains a first line treatment for severe pneumonia, septicaemia and meningitis locally (Steve Howie, personal communication) and is more affordable than cefotaxime or ciprofloxacin.

The predominance of serotype 1 clones such as ST618, that are not included in the current 7-valent conjugate vaccine, is of concern. Close surveillance of this clone is needed to monitor any possible increase after the introduction of vaccine. Changes over time after vaccination will, however, need to be interpreted with caution since we and others have shown substantial variation in incidence from year to year [2], and an increase after the introduction of vaccination may not necessarily indicate that replacement infection has occurred.

## Conclusion

For over a decade, isolates of ST618 have been the dominant lineage among serotype 1 carriage and disease isolates circulating in the Gambia. The existence of such an epidemic clone with stability in time and over geographical distance is interesting. ST618 also shows similar epidemiological features to those of the meningococcus in the African meningitis belt being able to cause outbreaks of disease.

## Authors' contributions

MA and RA conceived the study and wrote the paper with FC and BMG. MA and IH performed multiplex-PCR and analysis. OS, KS and DN cultured and identified bacteria isolates from clinical samples. GL, AA, UE, GE, PH, SMAZ, TC, BMG and FC participated in field and clinical aspects of the study. TA was involved in statistical analysis. All authors read and approved the final manuscript.

## References

[B1] Adegbola R, Hill P, Secka O, Ikumapayi U, Lahai G, Greenwood B, Corrah T (2006). Serotype and antimicrobial susceptibility patterns of isolates of Streptococcus pneumoniae causing invasive disease in The Gambia 1996–2003. Trop Med Int Health.

[B2] Leimkugel J, Adams Forgor A, Gagneux S, Pfluger V, Flierl C, Awine E, Naegeli M, Dangy J, Smith T, Hodgson A, Pluschke G (2005). An outbreak of serotype 1 Streptococcus pneumoniae meningitis in northern Ghana with features that are characteristic of Neisseria meningitidis meningitis epidemics. J Infect Dis.

[B3] Yaro S, Lourd M, Traore Y, Njanpop-Lafourcade B, Sawadogo A, Sangare L, Hien A, Ouedraogo M, Sanou O, Parent du Chatelet I, Koeck J, Gessner B (2006). Epidemiological and molecular characteristics of a highly lethal pneumococcal meningitis epidemic in Burkina Faso. Clin Infect Dis.

[B4] Dagan R, Gradstein S, Belmaker I, Porat N, Siton Y, Weber G, Janco J, Yagupsky P (2000). An outbreak of Streptococcus pneumoniae serotype 1 in a closed community in southern Israel. Clin Infect Dis.

[B5] DeMaria A, Browne K, Berk S, Sherwood E, McCabe W (1980). An outbreak of type 1 pneumococcal pneumonia in a men's shelter. JAMA.

[B6] Mercat A, Nguyen J, Dautzenberg B (1991). An outbreak of pneumococcal pneumonia in two men's shelters. Chest.

[B7] Hill P, Akisanya A, Sankareh K, Cheung Y, Saaka M, Lahai G, Greenwood B, Adegbola R (2006). Nasopharyngeal Carriage of Streptococcus pneumoniae in Gambian Villagers. Clin Infect Dis.

[B8] Bernatoniene JFA (2005). Advances in pneumococcal vaccines: advantages for infants and children. Drugs.

[B9] Cutts F, Zaman S, Enwere G, Jaffar S, Levine O, Okoko J, Oluwalana C, Vaughan A, Obaro S, Leach A, McAdam K, Biney E, Saaka M, Onwuchekwa U, Yallop F, Pierce N, Greenwood B, Adegbola R (2005). Efficacy of nine-valent pneumococcal conjugate vaccine against pneumonia and invasive pneumococcal disease in The Gambia: randomised, double-blind, placebo-controlled trial. Lancet.

[B10] Klugman K, Cutts F, Adegbola R, Black S, Madhi S, O'Brien K, Santosham M, Shinefield H, JAC S, Siber G, Klugman K (2008). Meta – analysis of the efficacy of conjugate vaccines against invasive pneumococcal disease. Pneumococcal Vaccines: the Impact of Conjugate Vaccines.

[B11] Antonio M, Dada-Adegbola H, Biney E, Awine T, O'Callaghan J, Pfluger V, Enwere G, Okoko B, Oluwalana C, Vaughan A, Zaman S, Pluschke G, Greenwood B, Cutts F, Adegbola R (2008). Molecular epidemiology of pneumococci obtained from Gambian children aged 2–29 months with invasive pneumococcal disease during a trial of a 9-valent pneumococcal conjugate vaccine. BMC Infect Dis.

[B12] Hill P, Cheung Y, Akisanya A, Sankareh K, Lahai G, Greenwood B, Adegbola R (2008). Nasopharyngeal Carriage of Streptococcus pneumoniae in Gambian Infants: A Longitudinal Study. Clin Infect Dis.

[B13] Adegbola R, Secka O, Lahai G, Lloyd-Evans N, Njie A, Usen S, Oluwalana C, Obaro S, Weber M, Corrah T, Mulholland K, McAdam K, Greenwood B, Milligan P (2005). Elimination of Haemophilus influenzae type b (Hib) disease from The Gambia after the introduction of routine immunisation with a Hib conjugate vaccine. Lancet.

[B14] http://www.ukneqas.org.uk/.

[B15] Enright M, Spratt G (1998). A multilocus sequence typing scheme for Streptococcus pneumoniae: identification of clones associated with serious invasive disease. Microbiology.

[B16] http://spneumoniae.mlst.net/.

[B17] http://eburst.mlst.net.

[B18] Brueggemann A, Spratt B (2003). Geographic distribution and clonal diversity of Streptococcus pneumoniae serotype 1 isolates. J Clin Microbiol.

[B19] Greenwood B (2006). 100 years of epidemic meningitis in West Africa – has anything changed?. Trop Med Int Health.

[B20] Zaman S (2008). Personal communication.

